# The Limits and Influences of a Nurse's Vocation

**Published:** 1888-01-28

**Authors:** R. Brudenell Carter

**Affiliations:** Member of the General Medical Council, Ophthalmic Surgeon to St. George's Hospital, and to the National Hospital for the Paralysed and Epileptic


### THE LIMITS AND INFLUENCES OF A NURSE'S VOCATION.

BY R. BRUDENELL CARTER, F.R.C.S.,

Member of the General Medical Council, Ophthalmic Surgeon to St. George's Hospital, and to the National Hospital for the Paralysed and Epileptic. A Lecture introductory to a Course on Nursing, delivered to the Nurses of the Chelsea Infirmary.—(*Continued from p.* 282.)

THERE are very few of the things which I have so far mentioned, which have not their bearing, direct or indirect, upon the comfort of the patient, and yet this comfort is sufficiently important to require a brief separate consideration. Our difficulties in dealing with the sick arise largely from the irritability of weakness, and from the repugnance which it produces to any effort or to any movement. If it should be necessary to give food often, and if the patient is indisposed to take it, how much will his dislike be increased by such a handling of the spoon or cup as may suffer the liquid to run down his chin and into his neck. How much will it be diminished if he finds, after a few trials, that the food is given tenderly and cleverly, and that he feels refreshed and invigorated when it has been taken. The same thing will apply to all changes of position, to all movements of the pillows or of the limbs, in short to all handling, which should always be done with a precise and definite idea, beforehand, of what the movement is to be and how it is to be accomplished. The making of the bed, the proper straining and the tucking in of the under sheet and under blanket, have an immense influence upon comfort, and, through comfort, upon the power of being quiet, or even of obtaining sleep; and the protection of the interior of the bed from crumbs is scarcely less important. If the medicine be nauseous, a little water to rinse it away from the mouth should be ready the instant it is swallowed, and should not have to be fetched from the other side of the room; and so on, with a score of seeming trifles which help to make up the enormous difference which exists between a good nurse and a bad one. There is a story of a great sculptor, whose friend, on visiting him, said that the statue on which he was engaged had made no progress since he, the friend, had seen it a month before. "Oh," said the sculptor, "I have done this and that. I have improved that fold of drapery, I have deepened that line on the face, I have given a bolder curve to that lip, I have modified the position of that hand. "Yes," said the friend," but all these things are trifles." "Perhaps so," answered the sculptor, "but the trifles, taken together, constitute perfection, and perfection is no trifle."

I have still to speak of watchfulness, concerning which I may perhaps say that it bears to the rest of good nursing the same relation as charity to the other Christian virtues, and that without it they are nothing worth. With a good nurse, the doctor, when he goes out of the sick room, feels that he has left his eyes behind him, and that when he comes again he will be told of every change which has occurred in the patient's condition. Without constant watchfulness, the nurse is little better than a machine. It is one thing to give food as soon as the patient's state requires it; it is quite another thing to give it when the clock strikes. All offices of this kind, all changes of position, all restorations of comfort, owe their efficacy to being timely, and they can be rendered timely by watchfulness alone. It is necessary not only that you should look at your patient, but that you should think about him as well; and that you should ask yourself, from time to time, what is the meaning of the changes which you witness.

And now, if you have given me your attention thus far, I shall hope to have convinced you, even by this slight glance at the duties you have undertaken to fulfil, that excellence as a nurse is indeed a thing difficult of attainment, but a thing which, at the same time, it is well worth taking trouble to attain. You will ask me, quite naturally and rightly, what is the end of it all, what are the rewards which you may expect. In the first place, you will always be able to secure the current rate of payment for skilled labour, whatever that may be; and my own impression is that it tends to increase rather than to diminish. I am very glad to say that a movement is on foot, and is likely to be carried to a successful issue, for providing a National Pension Fund for Trained Nurses, a fund to which you may subscribe during the period of your activity with the certainty of securing a provision in time of sickness, against infirmity, or in old age. If this can be secured, so that you may feel reasonably certain of remunerative employment and of ultimate support, let us think of the character of your industry. I judge of it by its likeness to my own, and I say that the kindly and skilful care of the sick carries with it the respect and esteem of all whose respect and esteem are worth possessing. I am old enough to remember when there were no trained nurses, and when the only people who could be procured to wait upon the sick were not only utterly ignorant of their duties, but scarcely ever made any intelligent attempt to understand them. As an instance of the watchfulness of those days I may tell you that, in the cholera epidemic of 1848, it was held by many doctors that the raging thirst of the patients must not be indulged, that is, that the drink for which they craved should be denied to them. A so-called nurse was obtained for a young man who was seized with cholera in his lodgings. She had an easy chair taken into his bed-room, told the servant of the house to bring up two pails of water, which would be wanted for washing, as the patient would be dead before morning, and then settled herself to sleep. When she awoke, the patient had emptied the pails, and was so much better as to be practically out of danger. The experience thus gained modified the treatment of cholera; but what are we to think of the nurse? In our great hospitals, if I may judge from that in which I was a student thirty-eight years ago, the head nurses of the wards were, indeed, women of good character, of much kindliness, and of some skill and knowledge acquired by long practice; but the assistant nurses, as they were called, were mere drudges, who were constantly being replaced by others, and who belonged mostly to the class of inferior domestic servants, whose conduct had been such as to place difficulties in the way of their being received into a family. If we turn from this picture to think of the estimation in which the trained nurses of the present day, some of whom have been honoured by receiving badges from the Queen herself, are held by all who employ or know them, the contrast, great as it is, is only a proper recognition of the change which has taken place in the facts. I was much amused a while ago by taking a doctor to a private hospital at which I do many operations, in order that he might select a bedroom for one of his patients who was about to go there for treatment. The mistress of the house was absent, and we were shown the rooms by one of the nurses. As the front door closed behind us, my friend stood on the doorstep and exclaimed, in words which he could contain no longer, "That is the cleanest-looking woman I ever saw in my life." The compliment was well-deserved, but was it not a great one? Do we not all know that a perfectly clean woman must have many other virtues besides cleanliness, and that her mind and thoughts are likely to be as clean and wholesome as her body? There is an old English proverb, often misquoted, which bears on this point. People say that "cleanliness is next to godliness," but the original word was "goodliness," which is an old-fashioned term for beauty. The proverb meant, indeed, that those who, by no fault of their own, could not be beautiful, might at least be clean; and, as I have given cleanliness the first place among the duties of a nurse, so I should give it the first place in her personal adornment. It should be her uniform and badge of office, and it will be admired wherever it is seen.

The work itself, often hard, always arduous and difficult, is nevertheless full of consolations. Men and women are so constituted as to feel pride and satisfaction in doing that which they have learnt to do well, and to derive from this source a content which is not far from being the best and truest form of happiness. Those of you whose work is among the rich have little to do beyond the control of the sick-room; but those who work among the poor may be, each one, and in some degree even must be, the silent but effectual teachers of a great reform. Is it possible that any of your poor patients in this building can leave it without having gained some appreciation of the advantages of cleanliness and order, of the importance of self-control, of the beauty of gentleness and kindness, of the excellence of the soft word which soothes irritability and impatience? Is not a perception of these things the first step towards some attempt to practise them; and are not your lives here to be regarded as constant examples to many who oftentimes stand much in need of the good qualities which your duties require you to display? In such a place as this, you can never tell where the good seed of such example may fall, or what fruit it may bear, but the most manifest opportunities of thus doing good offer themselves, I think, to those nurses who are retained to live in villages, and to nurse there all who may require them. A few weeks ago I was called to a village fifty miles from London, where I had to perform an operation. The ordinary servants of the house would have been unprepared for such an emergency; but, while I was sending for instruments and other necessaries, the "village nurse" arrived, and quickly reduced everything to order. I could not help thinking that the effect of her ministrations, which, in this case, were in a mansion, would have been still greater and more serviceable in a farmhouse or a cottage. I could not help feeling, as I watched her deft and handy ways, that she must be a source of neatness and of order wherever her influence was felt, and that the whole tone of the place in which she worked would be elevated by her example.

And I think there is yet one more consideration, and that not a small one, namely, that to be conversant with sickness and with death is a great help to us in arriving at a proper estimate of the real value of the prizes and distinctions of the world. We—doctors and nurses—see men and women as they are; we see that they are all pretty much alike, that when we know them well, and do not expect too much from them, they are all in many ways loveable, and that happiness is very equally divided between the palace and the cottage. The reflections which are thus forced upon us are, indeed, very much the same as those which have been left by a great author of the last century, Mr. Addison, in his account of the effect upon his mind of a visit to Westminster Abbey. I have had no recent opportunity of looking at his words, but I will quote them as nearly as I can from memory. "When," he says, "I stand before the sepulchres of the great, every emotion of envy dies within me; when I gaze at the tombs of the beautiful, every inordinate desire goes out; when I witness the grief of parents over their children, my heart bleeds with compassion; and when I see the monuments of the parents themselves, I am struck by the folly of sorrowing over those whom we must so shortly follow. And finally, when I consider the tombs of all periods, of some who died yesterday and of some who died five hundred years ago, I think of that great day when we shall all be contemporaries, and shall make our appearance together."

